# Efficient gene–environment interaction testing through bootstrap aggregating

**DOI:** 10.1038/s41598-023-28172-4

**Published:** 2023-01-17

**Authors:** Michael Lau, Sara Kress, Tamara Schikowski, Holger Schwender

**Affiliations:** 1 Mathematical Institute, Heinrich Heine University, Düsseldorf, Germany; 2 IUF – Leibniz Research Institute for Environmental Medicine, Düsseldorf, Germany

**Keywords:** Genetics, Environmental sciences, Diseases, Risk factors, Mathematics and computing, Computational science, Software, Statistics

## Abstract

Gene–environment (GxE) interactions are an important and sophisticated component in the manifestation of complex phenotypes. Simple univariate tests lack statistical power due to the need for multiple testing adjustment and not incorporating potential interplay between several genetic loci. Approaches based on internally constructed genetic risk scores (GRS) require the partitioning of the available sample into training and testing data sets, thus, lowering the effective sample size for testing the GxE interaction itself. To overcome these issues, we propose a statistical test that employs bagging (bootstrap aggregating) in the GRS construction step and utilizes its out-of-bag prediction mechanism. This approach has the key advantage that the full available data set can be used for both constructing the GRS and testing the GxE interaction. To also incorporate interactions between genetic loci, we, furthermore, investigate if using random forests as the GRS construction method in GxE interaction testing further increases the statistical power. In a simulation study, we show that both novel procedures lead to a higher statistical power for detecting GxE interactions, while still controlling the type I error. The random-forests-based test outperforms a bagging-based test that uses the elastic net as its base learner in most scenarios. An application of the testing procedures to a real data set from a German cohort study suggests that there might be a GxE interaction involving exposure to air pollution regarding rheumatoid arthritis.

## Introduction

Many complex diseases are influenced by both genetic and environmental risk factors. Often, their effects are studied individually, e.g., in genome-wide association studies (GWAS) or environmental health studies. These kinds of analyses, thus, study main/marginal effects of the respective risk factor type, i.e., effects independent of the other risk factor type. However, it is well known that the genetic make-up and environmental risk factors can influence the risk of disease in an interplay^1^. This phenomenon is known as gene–environment (GxE) interaction and is present if, for different genotypes, different disease susceptibilities to an environmental factor are underlying. This is, for example, the case if an individual is particularly susceptible to certain environmental exposure if the individual carries a specific genetic variant. For example, if an individual has xeroderma pigmentosum—a genetic defect that decreases the ability to repair DNA damage caused by ultraviolet radiation—and is exposed to sunlight, the risk effect of developing skin cancer through sun radiation is magnified compared to individuals without this genetic defect^1^.

Unveiling GxE interactions leads to a better understanding of the manifestation of complex diseases. Moreover, knowing specific GxE interactions could have a high impact on precision medicine by specifically protecting individuals that are highly susceptible to certain environmental health effects, i.e., performing individual risk prevention^2^.

In practice, GxE interactions are tested using single SNPs (single nucleotide polymorphisms—counting the number of less frequent base-pair substitutions at a specific locus in the DNA) in linear or logistic regression models. However, testing single SNPs in parallel requires adjustment for multiple testing, which reduces the statistical power for detecting a GxE interaction. To avoid this problematic, a GRS-(genetic risk score)-based approach can also be employed for taking multiple loci at once into account^3^. GRS summarize genetic variants with respect to a specific phenotype to a single statistic. Their utility can, e.g., lie in uncovering biological relationships in the development of diseases or their utility can be clinical for individual risk prevention^4–6^. GRS can be constructed internally—by using the considered study sample also for constructing the GRS—or externally—by using summary statistics of independent association studies. However, the external approach requires the availability of such summary statistics that match the considered outcome, the analyzed genomic region, and the considered population type, which might not be the case^3^. Moreover, by externally constructing GRS, only marginal genetic effects are considered, i.e., ignoring potential gene-gene interaction effects. Thus, we focus on the internal GRS construction approach in this article.

The GRS itself does not take any non-genetic variables into account. Thus, the variable which is used for interaction testing with the environmental term, the GRS, is a summary of genetic effects with respect to the outcome. However, for detecting GxE interactions, usually a GLM (generalized linear model) is fitted including potential confounders. For statistically testing the GxE interaction, typically a Wald test is employed.

The GRS approach leads to a major short coming. If an appropriate GRS model is not known beforehand, the available study data needs to be separated into two independent sub data sets, training data for constructing the GRS and test data for testing the GxE interaction. Therefore, the GxE interaction test cannot utilize the full available sample size, which reduces the statistical power for detecting a GxE interaction.

Several GxE interaction testing approaches have been proposed recently that avoid this data splitting problem^7,8^. Similar to the common GRS-based approach, SBERIA^9^ (set-based gene–environment interaction test) constructs a weighted sum of SNPs for testing the GxE interaction. Another class of GxE interaction tests is given by variance component tests that test the variance of the true interaction coefficients. The interaction effects are identified with random effects such that testing whether the interaction effect coefficients are zero is equivalent to testing if the underlying effect variance is zero^7^. Established methods of this class include GESAT^10^ (gene–environment set association test), iSKAT^11^ (interaction sequence kernel association test), and MiSTi^12^ (mixed effects score tests for interaction). Two-step GxE interaction testing procedures screen all considered SNPs and aggregate the positively screened SNPs to perform a global test for the presence of a GxE interaction among the considered SNPs in a second step^8^. The GxE interaction testing methods ADABF^13^ (adaptive combination of Bayes factor method), EDGxE^14^ (EG [environment-genotype] and DG [disease-genotype] screening with GxE interaction testing) and cocktail GxE interaction tests^15^ are such two-step procedures.

In this article, we propose a GxE interaction testing approach that overcomes the data splitting problem while being able to model arbitrarily complex genetic effects and avoiding the multiple testing problem of the single-SNP-based test. Similar to the classical GRS-based test, our test also relies on modeling the genetic effect on the outcome through a GRS. Our approach can incorporate the full study data set for both training the GRS and testing the GxE interaction while still avoiding the overfitting problem. The idea consists of constructing the GRS via the ensemble method bagging (bootstrap aggregating)^16^ and using its well-known OOB (out-of-bag) prediction mechanism for creating unbiased predictions on the whole training data.

Moreover, standard GRS construction methods such as the elastic net^3,17^ can usually only incorporate marginal genetic effects and no gene-gene interaction effects. For example, it might be possible that the environment-response relationship is significantly altered only if specific genetic variants at two different loci are present at once, thus, leading to a GxE interaction involving a gene-gene interaction that cannot be covered by classical GRS approaches.

As prior analyses showed^6^, using random forests instead of elastic net leads to a higher predictive ability of the GRS. Thus, we also propose using random forests^18^ instead of elastic net as the GRS construction procedure in GxE interaction testing. This would yield a GxE interaction testing approach that is not restricted to simplifying assumptions on genetic effects and can incorporate every possible gene-gene interaction.

In this article, first, established GxE interaction testing procedures are discussed. Next, a novel testing approach based on bagging and OOB predictions is introduced. Moreover, an extension of this testing procedure using random forests is proposed. The methods are evaluated and compared to existing approaches in a simulation study that considers multiple realistic scenarios. As this analysis shows, the proposed testing procedures yield a higher statistical power than the reference testing procedures in many scenarios. Lastly, the methods are applied to a real epidemiological data set from a cohort study testing a GxE interaction involving air pollution regarding rheumatoid arthritis.

## Methods

In the following, two standard approaches and three recently proposed approaches for testing GxE interactions are discussed first. Afterwards, we propose two novel approaches that can be used to overcome the drawbacks of the initially discussed methods.

### Existing methods

First, existing methods for GxE interaction testing are discussed.

#### Single-SNP-based GxE interaction test

The GxE interaction test based on single SNPs tests each considered SNP independently for a GxE interaction^3^. That is, for each $\textrm{SNP}_j$, $j \in \lbrace 1,\ldots ,p\rbrace$, a GLM

1 $$\begin{aligned} g(\mathbb {E}[Y \mid \textrm{SNP}_j, E, \varvec{C}]) = \beta _0 + \beta _1 \textrm{SNP}_j + \beta _2 E + \beta _3 \textrm{SNP}_j \times E + \sum _{i=1}^m \gamma _i C_i \end{aligned}$$

is fitted, where also potential confounders $$\varvec{C} = \begin{pmatrix} C_1&\ldots&C_m \end{pmatrix}$$ are included in the model to adjust the main effects of $\textrm{SNP}_j$ and the environmental variable *E* as well as their interaction effect for these variables. If a binary disease status is the considered outcome, logistic regression models via the link function $g = \textrm{logit}$ are fitted. If the considered outcome is continuous, the identity link $g = \textrm{Id}$ is used for fitting linear regression models.

In each of these models, the statistical hypothesis $H_0: \beta _3 = 0$ versus $H_1: \beta _3 \ne 0$ is tested, i.e., whether there is an interaction effect of the SNP and the environmental variable *E* on the outcome. A Wald test is usually performed for testing this hypothesis. Alternatively, a score test or a likelihood-ratio test can be carried out for testing the same hypothesis^19^. If, e.g., it should be tested whether a gene interacts with *E*, its SNPs are tested and the test decision for the whole gene is made by adjusting the individual SNP testing results for multiple testing. Usually, a Bonferroni correction is carried out^3,20^. If, after the Bonferroni correction, for at least one SNP the null hypothesis could be rejected, the global null hypothesis of no GxE interaction on the gene is rejected as well.

This approach has the advantage of not having to train a model but to directly perform statistical testing. Moreover, it is very simple, straightforward, and computationally feasible, since the individual tests can also be parallelized. However, due to considering individual SNPs and performing adjustment for multiple testing, statistical power for detecting a GxE interaction is lost.

#### GRS-based GxE interaction test

In contrast to the single SNP test, the GRS-based GxE interaction test aggregates multiple SNPs into one model and uses this model to test if there is an interaction in the considered genomic region^3^. Usually, the GRS is a linear combination of SNPs

2 $$\begin{aligned} \widehat{\textrm{GRS}} = {\hat{\alpha }}_0 + {\hat{\alpha }}_1 {\textrm{SNP}}_1 + \ldots + {\hat{\alpha }}_p \textrm{SNP}_p \end{aligned}$$

that is either constructed internally or externally.

External GRS rely on summary statistics of independent studies and use the individual SNP effect sizes for determining their weights ${\hat{\alpha }}_1, \ldots , {\hat{\alpha }}_p$^21,22^. This approach, thus, requires the availability of appropriate study data, i.e., the same outcome, the same genomic region, and the same population type had to be analyzed^23^. Furthermore, the external approach only allows the construction of linear GRS, i.e., in general not taking interactions between genetic loci into account.

Alternatively, GRS can be constructed internally^3,24^, which means that the available data has to be divided into independent training and test data sets. The GRS is constructed using the training data and evaluated on the test data. This data splitting is crucial to avoid overfitting, i.e., to avoid detecting effects that are solely made up of statistical noise and are recognized due to the model adapting to this statistical noise. The internal approach also allows to generalize the task of constructing GRS to a statistical learning problem, in which a function is to be fitted that maps the SNPs to the outcome and that does not necessarily have to be linear^6^.

When internally constructing GRS, usually a GLM-based procedure such as the elastic net^25^ is utilized^17,26^. The elastic net also fits a linear model (2)—yielding the weights ${\hat{\alpha }}_1, \ldots , {\hat{\alpha }}_p$ and intercept ${\hat{\alpha }}_0$—and regularizes the effect coefficients $$\varvec{\alpha } = \begin{pmatrix} \alpha _1&\ldots&\alpha _p \end{pmatrix}$$. This is done by including the penalty term

$$\begin{aligned} R_\xi (\varvec{\alpha }) = \frac{1}{2} (1-\xi ) ||\varvec{\alpha }||_2^2 + \xi ||\varvec{\alpha }||_1 \end{aligned}$$

in the optimization problem

$$\begin{aligned} \min _{\alpha _0, \varvec{\alpha }} \left\{ -\frac{1}{N} \ell (\alpha _0, \varvec{\alpha }) + \lambda R_\xi (\varvec{\alpha }) \right\} , \end{aligned}$$

in which $\ell$ is the log-likelihood function of the considered parameters, $\lambda$ is the penalty strength, and $\xi \in [0,1]$ is a parameter controlling the balance between the $L_1$ penalty and the $L_2$ penalty, i.e., the lasso penalty^27^ and the ridge penalty^28^, respectively. The lasso penalty leads to shrinking the coefficients of unimportant SNPs to zero while the ridge penalty assigns similar weights to highly correlated SNPs, which, e.g., might be the case for SNPs in high LD (linkage disequilibrium). Thus, the elastic net simultaneously performs a variable selection and a properly handling of SNPs in high LD. However, as for GLMs, only marginal SNP effects are modeled if no prior knowledge about which loci might interact is available, which is usually the case.

After constructing the GRS on training data, predictions $\widehat{\textrm{GRS}} = {\hat{\alpha }}_0 + {\hat{\alpha }}_1 \textrm{SNP}_1 + \ldots + {\hat{\alpha }}_p \textrm{SNP}_p$ on independent test data are performed. These predicted values of the GRS for the subjects in the test data set are then used to fit the GLM

3 $$\begin{aligned} g(\mathbb {E}[Y \mid \widehat{\textrm{GRS}}, E, \varvec{C}]) = \beta _0 + \beta _1 \widehat{\textrm{GRS}} + \beta _2 E + \beta _3 \widehat{\textrm{GRS}} \times E + \sum _{i=1}^m \gamma _i C_i. \end{aligned}$$

As for the single-SNP-based test, if a binary disease status is the phenotype of interest, the $\textrm{logit}$ is used as link function *g* for fitting logistic regression models in both the GRS construction step and the GxE testing step. For continuous phenotypes, linear regression models are fitted using the identity as link function *g*.

For testing if the considered genomic region interacts with *E* regarding the outcome *Y*, the statistical test $H_0: \beta _3 = 0$ versus $H_1: \beta _3 \ne 0$ is performed. Similar to the single-SNP-based test, this hypothesis is most commonly tested using a Wald test. A GxE interaction is present if $H_0$ is rejected at a prespecified level of significance. In contrast to the single-SNP-based test, this test result directly reflects the desired test decision such that no adjustment for multiple testing has to be performed.

The drawback of this GRS-based testing approach is the requirement for splitting the available data into independent training and test data sets, where simulation studies suggest that a random 50:50 split should be used^23^. However, since in this case only 50% of the data can be used for actually testing the GxE interaction, substantial statistical power for detecting the GxE interaction is lost.

#### Set-based gene–environment interaction test

SBERIA^9^ is a GxE interaction test that also utilizes a weighted sum of SNPs, similar to the GRS-based procedure. In SBERIA, all SNPs are univariately screened for either the association with the environmental factor or the association with the outcome. The results of this screening are used for constructing a weighted sum of SNPs. More precisely, this sum is constructed as $\widehat{\textrm{GRS}} = w_1 \textrm{SNP}_1 + \ldots + w_p \textrm{SNP}_p$ with

$$\begin{aligned} w_j = \varepsilon + {\left\{ \begin{array}{ll} 0, &{} \text {if}\,p\,\text {value for}\,\textrm{SNP}_j > \theta \\ -1, &{} \text {if}\,p\,\text {value for}\,\textrm{SNP}_j \le \theta \,\text {and correlation of}\, \textrm{SNP}_j \,\text {with}\,E\,(\text {or}\,Y) \,\text {negative} \\ +1, &{} \text {if}\,p\,\text {value for}\,\textrm{SNP}_j \le \theta \,\text {and correlation of}\, \textrm{SNP}_j \,\text {with}\,E\,(\text {or}\,Y) \,\text {positive}. \end{array}\right. } \end{aligned}$$

The offset $\varepsilon$ is usually chosen as 0.0001 and the significance threshold $\theta$ as 0.1. The GxE interaction is then tested as in the GRS-based test using the GLM from Eq. (3). However, in contrast to the GRS-based test, the weighted sum utilized in SBERIA only considers the magnitude of the genetic effects to a limited extent. Nonetheless, through this limited modeling, the overfitting problem of the GRS-based testing does not arise such that the full data can be utilized for constructing the weighted sum and testing the GxE interaction even in low sample size scenarios.

#### Gene–environment set association test

GESAT^10^ is a GxE interaction test that belongs to the class of variance component tests. In variance component tests, the GLM

$$\begin{aligned} g(\mathbb {E}[Y \mid \textbf{SNP}, E, \varvec{C}]) = \delta _0 + \varvec{\delta }_1^T \textbf{SNP} + \delta _2 E + \varvec{\delta }_3^T \textbf{SNP} \times E + \sum _{i=1}^m \gamma _i C_i \end{aligned}$$

is considered for testing the GxE interaction, where $$\textbf{SNP} = \begin{pmatrix} \textrm{SNP}_1&\ldots&\textrm{SNP}_p \end{pmatrix}$$ is the vector of all considered SNPs, $\varvec{\delta }_1$ is the vector of corresponding main effects and $\varvec{\delta }_3$ is the vector of corresponding GxE interaction effects. The GxE interaction effects are modeled as random effects with mean zero and a common variance $\tau \ge 0$. Testing the presence of a GxE interaction anywhere in the considered set of SNPs is now equivalent to testing $H_0: \tau = 0$ versus $H_1: \tau > 0$. In GESAT, a score test is used for testing this hypothesis. For computing the score test statistic, the main effects have to be estimated under the null model only incorporating main effects. In GESAT, this is done by applying ridge regression. The authors have shown that the score test statistic follows—under the null distribution of no GxE interaction—asymptotically a mixture of $\chi ^2$-distributions.

#### Adaptive combination of Bayes factor method

ADABF^13^ is a recently proposed GxE interaction testing approach that tries to overcome the issues of classical tests, i.e., the need for data splitting or for too conservative multiple testing adjustment, by considering Bayes factors. ADABF starts by individually screening all considered SNPs for associations with the outcome. Only the $p_S \le p$ SNPs passing this screening (e.g., only SNPs that are significantly associated with respect to a level of significance of 5%) are used for testing the GxE interaction itself. Similar to the single-SNP-based test, individual GLMs (see Eq. (1)) are fitted for each considered SNP. Then, Bayes factors

$$\begin{aligned} \textrm{BF} = \frac{\mathbb {P}(\textrm{data} \mid H_{1})}{\mathbb {P}(\textrm{data} \mid H_{0})} \end{aligned}$$

are computed for each SNP and the corresponding hypothesis $H_0: \beta _3 = 0$ versus $H_1: \beta _3 \ne 0$ of the GxE interaction coefficient of this SNP. Prior knowledge from previous studies is used for configuring the variance of the prior distributions of both the main effects and the GxE interaction effects. Since it is of interest to test the whole considered set of SNPs for a GxE interaction and not just single SNPs, the Bayes factors are combined into summary scores

$$\begin{aligned} S_k = \sum _{l=1}^k \log (\textrm{BF}_{(l)}) \end{aligned}$$

with $\textrm{BF}_{(l)}$ ($l \in \lbrace 1,\ldots ,p_S \rbrace$) being the decreasingly sorted Bayes factors for the considered SNPs such that $\textrm{BF}_{(1)} \ge \ldots \ge \textrm{BF}_{(p_S)}$. These summary scores are also computed under the null distribution of no GxE interaction, i.e., by randomly sampling GxE interaction effects from a multivariate normal distribution with mean zero (corresponding to no effect) and a covariance matrix incorporating LD (linkage disequilibrium) between the SNPs. Afterwards, the original summary scores and the sampled versions are compared for deriving *p* values for every $k \in \lbrace 1,\ldots ,p_S \rbrace$. Minima of these *p* values are computed for deriving a final *p* value that tests the global null hypothesis of no GxE interaction across all considered SNPs.

#### Bootstrap aggregating

To overcome the loss in statistical power through limited modeling or data splitting, we propose employing bagging (bootstrap aggregating)^16^ for constructing the GRS in GxE interaction testing. Bagging is an ensemble approach that constructs *B* single models and combines them to one prediction model by averaging over the predictions of the individual models. The number of models *B* is chosen prior to fitting the model and should be set to a sufficiently high number such that more iterations do not considerably change the ensemble model. Each individual model is fitted by randomly drawing a bootstrap sample from the complete available data set, i.e., drawing *N* observations with replacement from a data set consisting of *N* observations, and using this sample to train the model such as a GLM via elastic net. A key property of bagging is that it reduces the variance of the predictions, thus, stabilizing the predictions^29^.

Since in every iteration a bootstrap sample is used for training the model, there is complementary data left that was not used for training this sub model. These data are called OOB (out-of-bag) data. Utilizing this fact, unbiased predictions on the complete data set can be made. For each observation, those models are gathered that did not use this observation for training. These models are used to temporarily construct an ensemble and the OOB prediction is generated by calculating the average over these models, so that for an observation $(\varvec{x}, y)$, its OOB prediction ${\hat{y}}_{\textrm{OOB}}$ is calculated by

$$\begin{aligned} {\hat{y}}_{\textrm{OOB}} = \frac{1}{|\mathcal {F}_{(\varvec{x}, y)}|} \sum _{f \in \mathcal {F}_{(\varvec{x}, y)}} f(\varvec{x}) \end{aligned}$$

where

$$\begin{aligned} \mathcal {F}_{(\varvec{x}, y)} = \left\{ f \in \mathcal {F} \mid (\varvec{x}, y) \notin T_f \right\} \end{aligned}$$

is the set of all trained models that did not use the considered observation for training, $\mathcal {F}$ is the set of all trained models in the ensemble, and $T_f$ is the training data set used for training *f*. Thus, the OOB prediction for each observation is constructed by models that never have seen this specific observation, resembling test data predictions.

### Proposed methods

In the following, two novel GxE interaction testing methods based on bagging are introduced.

#### GxE interaction testing through bagging

For avoiding the data splitting problem in GxE interaction testing, we propose constructing the GRS using bagging, e.g., bagging using the elastic net as the base learner, and computing the OOB prediction for all individuals in the whole data set. These predictions can then be used as a predictor in the GLM (3) as before and the statistical hypotheses $H_0: \beta _3 = 0$ versus $H_1: \beta _3 \ne 0$ are tested using a Wald test analogously to the conventional GRS-based test. Note that in contrast to the conventional GRS-based test, the GLM (3) is fitted and tested using all available data. Similarly, the GRS is in this case also fitted using all available observations. Therefore, neither the modeling step nor the testing step suffer from reduced sample sizes in this approach.

**Figure 1:**
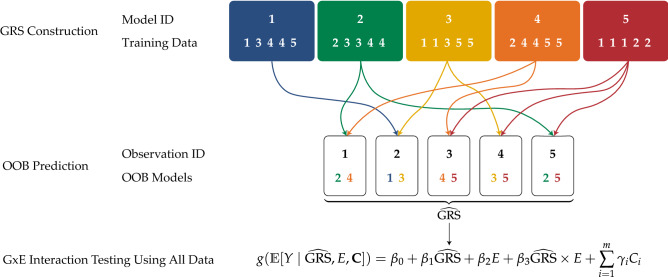
Exemplary GxE interaction testing workflow utilizing bootstrap aggregating. $N=5$ observations and $B=5$ bagging iterations are considered.

Figure 1 illustrates the proposed bagged GxE interaction testing approach considering $N = 5$ subjects and $B = 5$ bagging iterations/bootstrap samples (note that both numbers should actually be much higher; here, we only consider these small numbers for illustration purposes). First, for each out of $B=5$ bagging iterations, a bootstrap sample is drawn from the original sample consisting of $N = 5$ observations and used for training the respective model such as a GLM through elastic net. For example, in the first iteration, the model is fitted using the observations 1, 3, 4, and 5. Next, for each of the $N=5$ observations, those models are selected that did not use the respective observation for training and are used for generating the OOB predictions for the GRS. For example, for the first observation, the models 2 and 4 are used to predict its GRS by averaging their predictions, since observation 1 was not used for fitting the models 2 and 4. These predicted GRS values are then used as the values of the predictor to fit the GLM (3) and test whether the GxE interaction is associated with the outcome via its coefficient $\beta _3$ using a Wald test.

The GRS can be an arbitrary summary of genetic loci. Hence, if loci from multiple genes should be tested in a single GxE interaction test, the bagged GRS is constructed using all considered loci at once. For example in the real data application, a GxE interaction is tested for an association-based SNP selection that can potentially lead to loci from multiple different genes and for a gene-based SNP selection that was derived by considering multiple genes at once.

#### Random-forests-based GxE interaction test

Common GRS construction procedures such as the elastic net rely on linear modeling of genetic effects. Thus, these approaches usually model only marginal genetic effects unless prior knowledge about which loci might interact is available. Instead, more flexible modeling techniques such as random forests^18^, which are theoretically able to model every possible interaction, can also be used to construct GRS. It has been previously shown^6^ that these predictions can substantially outperform the standard method elastic net in the construction of GRS.

Therefore, we propose using random forests for the GRS construction step in testing GxE interactions. Random forests is an extension of bagging that uses decision trees^30^ as its base learner. The individual decision trees are further randomized by selecting random subsets of the predictor set in the recursive fitting procedure. This additional randomization leads to an increased variance reduction. Due to employing bagging, random forests is a natural candidate for applying the OOB-predictions-based GxE interaction test discussed in the previous section. Here, the sub models that are used for computing OOB predictions are the individual randomized decision trees.

### Ethics approval and consent to participate

The study was conducted in accordance to the declaration of Helsinki. The SALIA cohort study has been approved by the Ethics Committees of the Ruhr-University Bochum and the Heinrich Heine University Düsseldorf. Written informed consent was received from all participants.

## Simulation study

For examining the proposed GxE interaction testing procedures based on bagging using elastic net and on random forests, respectively, we compared these procedures with each other, with the two classical testing approaches, i.e., the single-SNP-based test and the GRS-based test using elastic net, and with three recently proposed GxE interaction testing procedures, namely ADABF, GESAT, and SBERIA, in a simulation study considering several realistic data scenarios.

### Simulation setup

In every simulation setting, 1000 independent replications were carried out, i.e., 1000 independent data sets were generated and evaluated for each considered study setting. The samples sizes were varied between $N = 500$, $N = 1000$, and $N = 2000$. 50 SNPs were simulated independently, resembling LD-based pruned SNPs, using random minor allele frequencies in the range of [0.15, 0.45], as in the analyses conducted by Lau et al.^6^. Similarly, dominant modes of inheritance were used for modeling the outcomes. The environmental term was generated by fitting a log-normal distribution on recorded exposures to nitrogen dioxide ($\textrm{NO}_2$) in the SALIA study^32^ and randomly sampling from this fitted log-normal distribution. The SALIA study is described in more detail in the following section, in which the data from this study is also used in a real data application.

For the GRS-based testing approach employing elastic net, the data sets have to be divided into training and test data sets. Random 50:50 splits were used as recommended by Hüls et al.^23^ in the context of GxE interaction testing. Additionally, a binary and a continuous outcome were simulated and analyzed. For the binary outcome, the prevalence, i.e., the probability of developing a disease without any exposure and genetic susceptibility, was chosen in each setting such that balanced data sets were generated, i.e., data sets, in which approximately half of the observations are cases and the other half are controls, which resembles (balanced) case-control studies.

The outcomes were generated following GLMs that are described in more detail below. Both the binary and the continuous outcomes used the same linear predictors. For the binary outcome, the inverse of the $\textrm{logit}$ link function was used to generate case probabilities $\mathbb {P}(Y = 1 \mid \textbf{SNP}, E)$ for randomly sampling the simulated outcome. For the continuous outcome, random noise from the standard normal distribution was added to each linear predictor $\mathbb {E}[Y \mid \textbf{SNP}, E]$.

#### Type I error

First, the type I error rate of the testing procedures was evaluated, i.e., the probability of falsely rejecting the null hypothesis, which in this case is the probability of detecting a GxE interaction although no GxE interaction is present. In all cases, the typically used level of significance of 5% was considered. We, thus, checked if the proposed tests control the type I error rate at a level of 5%.

For evaluating the type I error rate, data sets were simulated by considering the model

$$\begin{aligned} g(\mathbb {E}[Y \mid \textbf{SNP}, E]) = \alpha _0 + \alpha _1 \textrm{SNP}_{1,D} + \alpha _2 \textrm{SNP}_{2,D} + \alpha _3 \textrm{SNP}_{3,D} + \alpha _4 \textrm{SNP}_{4,D} + \alpha _{\textrm{GxG}} \textrm{SNP}_{1,D} \textrm{SNP}_{5,D} + \alpha _E E, \end{aligned}$$

where $\textrm{SNP}_{i,D} {:}{=} \mathbbm {1}(\textrm{SNP}_i > 0)$ is a SNP exhibiting a dominant mode of inheritance. In this model, thus, no GxE interaction is present. The marginal genetic effect sizes $\alpha _1 = \alpha _2 = \alpha _3 = \alpha _4 = \log (1.5)$ were fixed to an odds ratio of 1.5, resembling moderate effects. The gene-gene interaction effect was fixed to $\alpha _{\textrm{GxG}} = 2\log (1.5) = \log (2.25)$. The marginal environmental effect was fixed to $\alpha _E = \log (1.02)$. The effect size for the environmental term may seem rather small compared to the genetic effect sizes. However, this is due to the fact that the environmental factor in the SALIA study attains higher values with a median of 23.91.

#### Power—different GxE interaction effect intensities and sample sizes

Next, we evaluated the statistical power of the proposed GxE interaction tests, where the power is the probability of correctly rejecting the null hypothesis, i.e., the probability of detecting a true GxE interaction.

For evaluating the power, the model

4 $$\begin{aligned} \begin{aligned} g(\mathbb {E}[Y \mid \textbf{SNP}, E])&= \alpha _0 + \alpha _1 \textrm{SNP}_{1,D} + \alpha _2 \textrm{SNP}_{2,D} + \alpha _E E \\&\quad + \alpha _{\textrm{GxE}} \textrm{SNP}_{3,D} E + \alpha _{\textrm{GxE}} \textrm{SNP}_{4,D} E + 2\alpha _{\textrm{GxE}} \textrm{SNP}_{1,D} \textrm{SNP}_{5,D} E \end{aligned} \end{aligned}$$

was used for generating the data sets.

**Figure 2:**
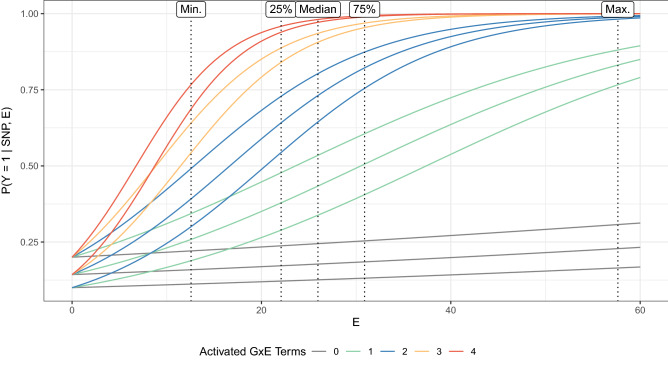
Design for simulating a binary phenotype. Exposure-response curves are depicted for different genotypes and a strong GxE interaction effect $\alpha _{\textrm{GxE}} = \log (1.05)$ in Eq. (4). The colors illustrate the GxE interaction intensity referring to the number of activated GxE interaction terms in Eq. (4). The utilized distribution of the environmental factor is shown at the top by the 25%, 50%, and 75% quantiles and the minimum and the maximum.

For choosing realistic parameters, we analyzed the true underlying models. The final parameter choice is illustrated in Fig. 2, which depicts the desired simulation setup through the true modeling probabilities $\mathbb {P}(Y = 1 \mid \textbf{SNP}, E)$. The three curves at the bottom almost resembling a linear relationship correspond to the case of only the marginal environmental effect being active, i.e., no interacting risk allele being present that increases the slope. Thus, these curves share the same slope. The curves above are induced by interacting risk alleles being present and increasing the slope. In this model, almost the whole range in the probability spectrum is covered, i.e., probabilities $\mathbb {P}(Y = 1 \mid \textbf{SNP}, E)$ of almost 100% if all risk alleles are present at once and a high exposure is given or probabilities $\mathbb {P}(Y = 1 \mid \textbf{SNP}, E)$ of around 10% if no risk alleles and no exposure are given.

The corresponding model parameters are, therefore, chosen as follows. As in the type I error evaluation, the marginal genetic effects were fixed to $\alpha _1 = \alpha _2 = \log (1.5)$. The marginal environmental effect size was fixed to $\alpha _E = \log (1.01)$ and the effect size $\alpha _{\textrm{GxE}}$ of the GxE interaction involving $\textrm{SNP}_3$ and $\textrm{SNP}_4$ was varied between $\log (1.01)$, $\log (1.03)$, and $\log (1.05)$. The effect size $2\alpha _{\textrm{GxE}}$ of the GxE interaction also incorporating a GxG interaction was doubled, since interaction effects are in general more difficult to capture, i.e., requiring more data or stronger effect sizes.

#### Power—main effects and different levels of statistical noise

For analyzing the methods’ performances under the presence or absence of main effects and different levels of statistical noise, further simulations were conducted. The simulation setup considered by Lin et al.^13^ was used as a basis for these simulations. Thus, the outcome was generated using the model

5 $$\begin{aligned} g(\mathbb {E}[Y \mid \textbf{SNP}, E]) = \sum _{j=1}^K \alpha _j \textrm{SNP}_{j,D}\ + \sum _{j=0.5K+1}^{1.5K} \alpha _{\textrm{GxE}_j} \textrm{SNP}_{j,D} E. \end{aligned}$$

Therefore, half of the interacting SNPs also exhibit main effects if the corresponding coefficients are unequal to zero. The number *K* of interacting SNPs and SNPs that may exhibit main effects was set to 10. The number of total SNPs was varied between 20, 50, and 100, simulating different settings of statistical noise by including more SNPs that have theoretically no effect on the outcome. The sample size was set to $N=2000$, since different samples sizes were already analyzed in the previously described simulation scenario. For every setting, 100 independent replications were conducted, i.e., 100 independent data sets were generated and evaluated for each considered setting in this additional simulation scenario. The SNPs were simulated analogously to the previous simulation scenario, i.e., independently (resembling LD-based pruned SNPs) and using random minor allele frequencies in the range of [0.15, 0.45]. Similar to Lin et al.^13^, the environmental variable *E* was generated as a binary variable with $\mathbb {P}(E=1)=\mathbb {P}(E=0)=0.5$.

Analogously to Lin et al.^13^, the GxE interaction testing procedures were evaluated in four different simulation settings. These simulation settings are summarized in Table 1. First, two settings with no main effects, i.e., $\alpha _j = 0$ for all $j \in \lbrace 1,\ldots ,K \rbrace$, were evaluated. In these two settings, the effect sizes were varied. In the first setting, the coefficients were randomly drawn from the uniform distribution on $[\log (1.2), \log (1.4)]$ for the binary outcome and from the uniform distribution on [0.13, 0.17] for the continuous outcome. These effect sizes resemble small genetic effects. In the second setting, larger genetic effects were included. Hence, coefficients from the uniform distribution on $[\log (1.4), \log (1.6)]$ for the binary outcome and from the uniform distribution on [0.18, 0.22] for the continuous outcome were randomly drawn in the second simulation setting. Furthermore, two settings including main effects were evaluated. Here, the main effect and GxE interaction effects were randomly drawn according to the previously described uniform distributions. In all settings, the signs of the effect coefficients were randomly drawn. Thus, settings in which the main effect and the corresponding GxE interaction effect point in the same direction are covered as well as settings in which the main effect and the corresponding GxE interaction effect point in different directions are covered.

**Table 1:** Simulation settings for the second simulation scenario (see Eq. (5)) considering different effect sizes, different levels of statistical noise, and the presence or absence of main effects.

Setting	Binary $\alpha _1, \ldots , \alpha _{10}$	Continuous $\alpha _1, \ldots , \alpha _{10}$	Binary $\alpha _{\textrm{GxE}_6}, \ldots , \alpha _{\textrm{GxE}_{15}}$	Continuous $\alpha _{\textrm{GxE}_6}, \ldots , \alpha _{\textrm{GxE}_{15}}$
1	0	0	$\pm [\log (1.2),\log (1.4)]$	$\pm [0.13, 0.17]$
2	0	0	$\pm [\log (1.4),\log (1.6)]$	$\pm [0.18, 0.22]$
3	$\pm [\log (1.2),\log (1.4)]$	$\pm [0.13, 0.17]$	$\pm [\log (1.2),\log (1.4)]$	$\pm [0.13, 0.17]$
4	$\pm [\log (1.4),\log (1.6)]$	$\pm [0.18, 0.22]$	$\pm [\log (1.4),\log (1.6)]$	$\pm [0.18, 0.22]$

### Application of the GxE interaction tests

The application of the GRS-based GxE interaction testing procedures requires the choice of reasonable parameter settings for the underlying statistical learning method.

For fitting elastic net models, the strength of the penalty $\lambda \ge 0$ has to be tuned, which is usually done by *k*-fold cross-validation. In this article, 10-fold cross-validation was employed throughout all analyses. The balance parameter $\xi$ was fixed to 0.5, as 0.5 is a reasonable value in most situations for constructing GRS^3^. The R^31^ software package glmnet^33^ was used for fitting elastic net models.

For the novel bagging-based GxE interaction tests, the number *B* of bagging iterations has to be chosen. In this article, we set $B = 500$, since this is a relatively high number of bagging iterations, such that more iterations would not considerably alter the ensemble.

For fitting random forests, the R software package ranger^34^ was used. For the number of random variables drawn for evaluating tree splits, the standard setting of random forests for a higher number of predictors was used, i.e., mtry was set to $\lfloor p/3 \rfloor$, where *p* is the number of SNPs. The minimum number of observations contained in a terminal node (min.node.size) was set to $\lfloor 0.05 \times n_{\textrm{tree}} \rfloor$, in which $n_{\textrm{tree}}$ is the number of observations one single tree uses for training. If bootstrap sampling is performed, $n_{\textrm{tree}} = N$, in which *N* is the total sample size, holds. This setting was used to avoid too deep trees that overfit and to fit trees that hold stable risk estimates in their leaves, as suggested by Malley et al.^35^. The number of trees in a random forest (num.trees) was set to 500, the standard setting in ranger.

ADABF tests were carried out using the standard settings and the corresponding code that is available online (https://homepage.ntu.edu.tw/~linwy/ADABFGE.html). GESAT tests were conducted utilizing the R package iSKAT (https://github.com/lin-lab/iSKAT-GESAT) using its standard settings. Due to lack of publicly available software, the SBERIA test was implemented manually and carried out using 0.0001 as the intercept for non-significant SNPs and 0.1 as the *p* value threshold, as proposed by Jiao et al.^9^.

### Results of the simulation study

In the following, the results of the simulation study are presented.

#### Type I error

**Figure 3:**
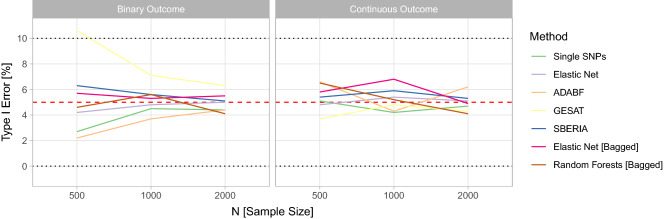
Type I error rates of the single SNP test, the GRS-based test using elastic net, ADABF, GESAT, SBERIA, the bagged GRS-based test using elastic net, and the random-forests-based test for testing GxE interactions in the simulation study.

Figure 3 shows the estimated type I error rates for the considered methodologies. The red dashed line indicates the targeted 5% level. Both the bagged test using elastic net and random-forests-based test induce type I error rates that are around this level for both binary and continuous outcomes and smaller to larger data sets. Thus, the proposed methods seem to control the type I error. Similarly, the reference testing procedures based on single SNPs and elastic net regression also yield type I error rates around the 5% level. The alternative GxE interaction testing approaches ADABF and SBERIA induce type I error rates around 5% as well. However, in our simulation study, GESAT yields a type I error rate of over 10% for small samples and a binary outcome. Also for larger sample sizes and a binary outcome, GESAT induces higher type I error rates than the other methods. This issue might be caused by asymptotics that have not been reached due to the small sample size but are vital to the theory of the GESAT test, in particular, for the distribution under the null hypothesis of no GxE interaction.

#### Power—different GxE interaction effect intensities and sample sizes

**Figure 4:**
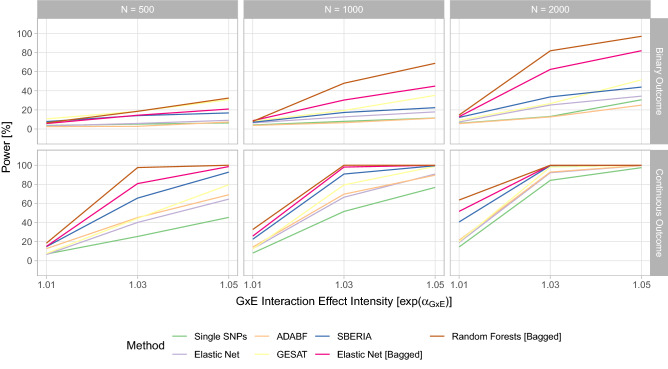
Statistical power of the single SNP test, the GRS-based test using elastic net, ADABF, GESAT, SBERIA, the bagged GRS-based test using elastic net, and the random-forests-based test for testing GxE interactions in the first scenario (see Eq. (4)) of the simulation study.

In Fig. 4, the results for the power evaluation of the first simulation scenario considering different GxE interaction effect intensities, different sample sizes, and a continuous environmental factor are shown. Unsurprisingly, the power rises with the available sample size and with the GxE effect intensity for all considered methods. For a large sample size and a strong GxE interaction effect, a power of 100% is reached, while for a small sample size and a weak GxE interaction effect, the power is around the prespecified tolerated type I error level. Therefore, the simulation design covers also scenarios in which the GxE interaction effect is almost undetectable and scenarios in which the GxE interaction should be detected, which was desired.

Regarding the comparison between the individual testing approaches, the single-SNP-based test seems to yield the lowest statistical power in most settings. For a continuous outcome, the GRS-based test, GESAT, and ADABF induce similar results. For a binary outcome, GESAT induces the highest power among these three tests. SBERIA yields the highest statistical power among the considered reference GxE interaction testing procedures.

The newly proposed proposed procedure based on bagging and using elastic net as its base learner consistently achieves superior results to the reference approaches. The novel random-forests-based method induces an even higher statistical power than the bagging-based test that uses elastic net.

Thus, regardless of considering a binary or a continuous outcome, small or large samples, or weak or strong GxE interaction effects, the two tests based on bagging induce a comparatively high statistical power.

#### Power—main effects and different levels of statistical noise

**Figure 5:**
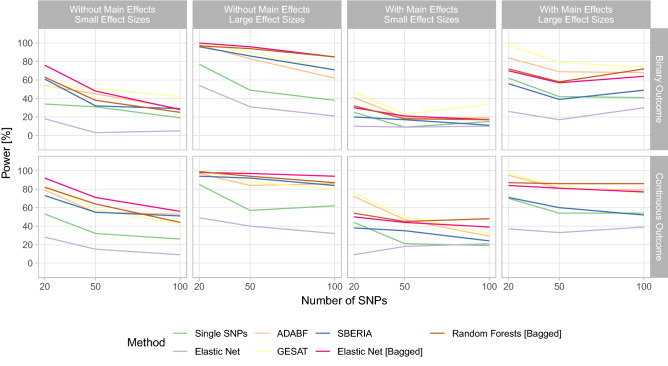
Statistical power of the single SNP test, the GRS-based test using elastic net, ADABF, GESAT, SBERIA, the bagged GRS-based test using elastic net, and the random-forests-based test for testing GxE interactions in the second scenario (see Eq. (5)) of the simulation study.

Considering the second simulation scenario analyzing different levels of statistical noise, the presence or absence of main effects, differing effect sizes, and a dichotomous environmental risk factor, Fig. 5 depicts the statistical power achieved by the considered GxE interaction testing methodologies. The GRS-based GxE interaction test employing elastic net and the single-SNP-based test induce the lowest statistical power in most settings. When no main effects are present, SBERIA seems to yield better results than the two classical testing approaches. GESAT and ADABF induce a similar statistical power that is higher than the statistical power induced by the other methods—including the proposed methods—when considering settings with main effects and a low number of additional noise SNPs. The proposed bagging-based testing approaches yield a comparatively high statistical power in all settings. When considering settings without main effects or settings with main effects and a higher number of noise SNPs, the power of the bagging-based tests is particularly high.

The bagging-based test employing elastic net as its base learner seems to induce a slightly higher power than the random-forests-based test when considering settings without main effects. Vice versa, with main effects, random forests yields a slightly higher power. In general, the induced statistical power by the bagging-based tests is similar in this simulation scenario. The bagging-based test with elastic net presumably yields a higher power in this scenario (see Eq. (5)) due to considering a pure linear relationship compared to the previous scenario (see Eq. (4)), in which a gene-gene interaction was also present.

The proposed bagging-based tests seem to be more robust against a higher number of noise SNPs, since their statistical power does not severely decrease in comparison to the other procedures. Only for the first setting without main effects and small effect sizes, their statistical power considerably decreases for a higher number of noise SNPs.

## Real data application

To verify the results of the simulation study and the applicability of the two proposed GxE interaction tests, a real data set from a German cohort study, the SALIA study (**S**tudy on the Influence of **A**ir Pollution on **L**ung, **I**nflammation and **A**ging)^32^, was used for investigating GxE interactions.

At baseline, the SALIA study was conducted between 1985–1994 and included 4874 women aged between 54 and 55 years at their baseline examination. The study region included highly and less industrialized areas in North Rhine-Westphalia, Germany. In 2006, 4027 study participants completed a follow-up questionnaire about the diagnosis of chronic diseases. A further follow-up involving clinical examinations was conducted in 2007–2010, where genetic data was collected. Genome-wide genotyping was performed using the Axiom Precision Medicine Research Array GRCh37/hg19 (Affymetrix, Santa Clara, CA, USA). Imputation of unobserved genotypes using the Haplotype Reference Consortium^36^ as reference panel on the Michigan Imputation Server^37^ and quality controls^38^ were performed. Individual exposures to air pollutants such as $\textrm{NO}_2$ during the first follow-up examinations were assessed using land-use regression models as part of the ESCAPE (European Study of Cohorts for Air Pollution Effects) project^39,40^.

In the questionnaire of the first follow-up examination, the study participants were asked if they had a diagnosed rheumatic disease. Therefore, prevalent rheumatic diseases were considered as outcome in this article. Among the 560 women, 144 women stated they had a diagnosed rheumatic disease so that 416 women stated they did not have a rheumatic disease. Since rheumatoid arthritis is the most common rheumatic disease besides osteoarthritis^41–43^, we focused on rheumatoid arthritis.

The data set analyzed in this article was restricted to subjects with available genotype data and information on the presence of rheumatic diseases. Thus, the analyzed data set consists of data from 560 women.

Gene ATLAS^44^ was used for selecting SNPs that are significantly associated with the development of rheumatoid arthritis in the UK Biobank^45^ (data field 20002). In particular, all SNPs that reached a level of significance of $10^{-80}$ were selected, which resulted in 91 SNPs in total. Canela-Xandri et al.^44^ computed these *p* values by performing two-sided t-tests for each SNP on the residuals of linear mixed-effects models that were fitted for each trait and include potential confounders such as sex or age as fixed effects and a random effect adjusting for the population structure. The significance threshold was chosen such that about 100 SNPs were selected. 87 of these 91 SNPs were available in the analyzed data set from the SALIA cohort study. A detailed list of the analyzed SNPs can be found in Supplementary Table S1. This first SNP selection is based on single SNPs that showed a significant association with the disease phenotype of rheumatoid arthritis.

Moreover, we also considered a gene-based SNP selection for confirming the applicability of the proposed GxE interaction tests in gene-based analyses. Analogously to Lau et al.^6^, the three genes HLA-DRB1, HLA-DPB1, and HLA-DOA from the human leukocyte antigen (HLA) class II complex were chosen, since they seem to explain a large fraction of the heritability of rheumatoid arthritis in the HLA class II complex^46–51^. All available SNPs from these three genes were selected, which resulted first in 385 SNPs. These SNPs were then clumped based on LD (linkage disequilibrium)^52^ considering $r^2 = 0.5$ using PLINK version 1.9^53^. The LD-based clumping resulted in 72 tag SNPs. This set of 72 gene-based selected SNPs and the set of 87 association-based selected SNPs are disjoint such that there is no single SNP that is present in both sets.

It has already been shown that an interaction between genetic risk factors and smoking exists in the development of rheumatoid arthritis^54,55^. Thus, it can be suspected that traffic-related air pollution such as $\textrm{NO}_2$ might also be involved in a GxE interaction, which is analyzed in the following. Hence, for testing the presence of a GxE interaction, we considered the exposure to $\textrm{NO}_2$ as the environmental variable potentially interacting with genetic risk factors.

Adjustment for relevant potential confounders was performed using the same set of potential confounders as Hüls et al.^56^ in their GxE interaction analysis. In particular, the genetic and environmental marginal effects as well as the GxE interaction effect were adjusted for subject age, socioeconomic status, BMI, smoking status, passive smoking, and household heating by indoor combustion of fossil fuels.

For more details about the SALIA study itself and an analysis of rheumatic diseases in the SALIA study, see Krämer et al.^57^ and Lau et al.^6^, respectively.

The evaluated GxE interaction methods were applied analogously to the simulation study. For application details, see Section “Application of the GxE interaction tests”.

### Results of the real data application

**Figure 6:**
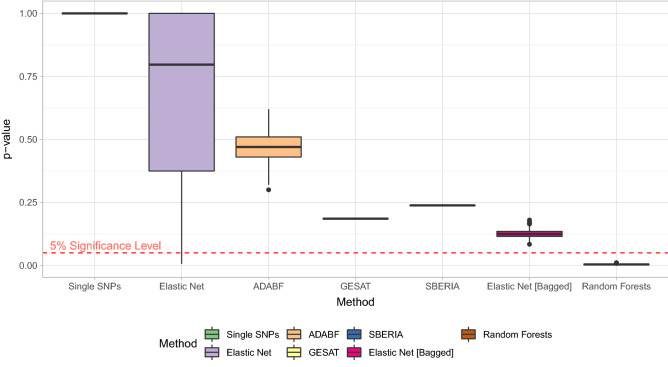
*p* values of 1000 independent applications of the GxE interaction testing procedures to the considered real data set from the SALIA cohort study analyzing the association-based SNP selection containing 87 SNPs. For elastic net, the train/test data splits changed. For the two bagging-based tests, the bootstrap samples changed. For ADABF, the random sampling from the null distribution of GxE interaction coefficients changed. The single-SNP-based test, GESAT, and SBERIA were applied only once, since there is no randomness involved in the application of these tests.

Figure 6 summarizes the results of the real data analysis considering the association-based SNP selection by the induced *p* values of the considered methodologies. The single-SNP-based test yields a *p* value of 1 due to the Bonferroni correction, i.e., none out of the 87 SNPs yields a significant GxE interaction. Without the Bonferroni correction, the single-SNP-based test would yield a *p* value of 0.125, which is still not significant with respect to a level of significance of 5%. However, note that not correcting for multiple testing would inflate the type I error rate, which would disqualify the single-SNP-based test as a valid statistical test.

The common GRS-based test employing elastic net yields a median *p* value of about 0.8 such that, in almost all repetitions, no GxE interaction was detected. However, the resulting *p* value heavily varies between the replications. This variance is induced by random data splits for training the GRS and testing the GxE interaction and by cross validation that randomly splits the respective training data set for choosing the ideal elastic net regularization penalty.

ADABF yields a median *p* value of 0.47—not indicating a GxE interaction.

GESAT and SBERIA induce *p* values of 0.19 and 0.24, respectively, which are substantially lower than the *p* values of the other reference testing procedures. However, the null hypothesis of no GxE interaction cannot be rejected with respect to a level of significance of 5%. Note that GESAT was not applied to the original set of 87 SNPs but to a LD-based pruned (using $r^2 = 0.975$) set consisting of 37 SNPs, since the GESAT software could not be applied to the original SNP selection, which seemed to be due to the very high LD of some of the SNPs.

The novel bagging-based test utilizing elastic net yields a median *p* value of about 0.12, which is not significant with respect to a level of significance of 5%, however, considerably lower than the median *p* value induced by the common tests. In no iteration, this test could detect a GxE interaction. Nonetheless, the variance of the resulting *p* values seems to be almost completely diminished in contrast to the common elastic-net-based test that does not employ bagging.

Using random forests, a median *p* value of about 0.004 is yielded, which is by far the lowest. In every repetition, the random-forests-based test rejects the null hypothesis of no GxE interaction. Thus, this suggests that there might be a GxE interaction between genetic risk factors and air pollution exposure regarding rheumatoid arthritis.

The results of the additional gene-based analysis are depicted in Supplementary Fig. S1. For these genes, none of the GxE interaction tests indicates the presence of a GxE interaction. In this analysis, random forests yields similar *p* values to ADABF and SBERIA, while the bagging-based test with elastic net as its base learner yields similar *p* values to GESAT and the single-SNP-based test.

## Discussion

In this article, we proposed a novel GxE interaction testing approach utilizing bagging and its OOB prediction mechanism. We further proposed using random forests as the GRS construction method in GxE interaction testing. The main advantage of these novel tests is that they allow to utilize all subjects in both the GRS construction and the GxE interaction testing in contrast to classical procedures. Furthermore, this general approach allows utilizing statistical learning procedures that can model more complex patterns such as decision trees in random forests.

The new methods were first compared to two commonly used procedures, the single-SNP-based test and standard GRS-based test, and three recently proposed procedures, ADABF, GESAT, and SBERIA, in a simulation study considering both binary and continuous outcomes as well as different sample sizes, GxE interaction effect sizes, different levels of statistical noise, and the presence or absence of main effects. The analyses were started by evaluating the type I error rate, i.e., the probability of detecting a false GxE interaction, to see whether the proposed methods are valid statistical tests. Both tests could control the type I error with respect to the prespecified significance level. The analyses were continued by evaluating the statistical power, i.e., the probability of detecting a true GxE interaction. Here, it could be observed that the proposed methods could induce strong results compared to the reference tests in most scenarios. In particular, for small sample sizes, in presence of gene-gene interactions, for high intensities of statistical noise, or in absence of main effects, the proposed tests induced a superior statistical power compared to the other considered tests. The random-forests-based test also yielded a considerably higher statistical power than the bagging-based test using elastic net as its base learner in most settings. In a real data application, a GxE interaction regarding rheumatoid arthritis involving the exposure to $\textrm{NO}_2$ was analyzed. The two novel methods induced the lowest *p* values. The random-forests-based test was the only test to consistently induce *p* values below the prespecified significance threshold, which suggests that there might be a GxE interaction.

The strength of the proposed tests can be largely explained due to the increased sample size for both constructing the GRS and testing the GxE interaction compared to the standard GRS-based test. However, the variance reducing property of bagging presumably also lead to improved GRS models that had a stronger association with the analyzed phenotype, which also likely increased the statistical power. A variance reduction could be seen in the real data application, where the bagging-based test using elastic net as its base learner considerably reduced the *p* value variance compared to the common elastic-net-based test without bagging. Here, the variance was reduced due to no longer requiring random train-test data splits and through bagging, reducing the variance induced by cross validation in fitting elastic net models. Moreover, the random-forests-based test’s superior performance was also caused by the ability to detect gene-gene interactions, which is usually not possible with elastic net, and the increased variance reduction due to further randomizing the model fitting procedure. In the second simulation scenario considering no gene-gene interactions, performances of both bagging-based tests were similar. Therefore, there seems to be no drawback of using the random-forests-based test over the bagging-based test using elastic net as its base learner. These findings are in line with the analyses by Lau et al.^6^, which showed that the predictive performance of a GRS constructed by random forests could compete with the predictive performance of a GRS constructed by elastic net, even when considering no gene-gene interaction effects.

The recently proposed GxE interaction testing approaches ADABF, SBERIA, and GESAT also do not rely on data splitting that is required by the conventional GRS-based GxE interaction test. However, our proposed bagging-based test offers the advantage of being able to capture arbitrarily complex genetic effects through the statistical learning procedure that is employed as base learner. In contrast to ADABF and SBERIA, our proposed methods do not explicitly perform variable selection before testing the GxE interaction. It might be that certain SNPs are excluded in the individual bagging iterations. However, these selections might only be valid for individual iterations and not for the ensemble model such that most, if not all, variables are most likely included in the GRS for testing the GxE interaction in some way. Nonetheless, due to the explicit regularization performed by elastic net or the implicit regularization performed by random forests through randomization^58^, possibly uninformative SNPs should not considerably decrease the statistical power, as could be seen in the simulation study.

As discussed by Janitza and Hornung^59^ and Mitchell^60^, the OOB error in random forests can be biased in the sense that it overestimates the actual test error. To eliminate this bias, they suggest to perform subsampling without replacement instead of bootstrap sampling. In this case, the number of observations in a subsample drawn is set to about $0.632 \times N$, the asymptotic number of unique observations drawn when performing bootstrapping. For evaluating if sampling without replacement would further improve the performance of our proposed GxE interaction testing procedures, we repeated the analyses with sampling without replacement. The results are shown in Supplementary Fig. S2–S4 and are in line with the evaluations using sampling with replacement. Hence, no considerable difference could be observed.

With GxE interaction tests that perform a SNP selection prior to testing the GxE interaction itself such as the single-SNP-based test, the GRS-based test employing elastic net or the lasso, ADABF, or SBERIA, it is relatively simple to deduce which SNPs among all initially considered SNPs are likely to be responsible for a detected GxE interaction. With the bagging-based approach, the GRS becomes an ensemble of many models such that it is not obvious how to infer the subset of SNPs responsible for a detected GxE interaction. Nonetheless, in future research, the proposed methodology could be extended to be able to score which genetic loci influence the constructed model the most, e.g., by employing VIMs (variable importance measures).

In our evaluations, we used fixed hyperparameter settings for fitting random forests. However, especially the parameter for determining the number of random variables selected as potential splitting variables and the parameter for bounding the minimum number of observations contained in a single leaf can have a substantial impact on the performance of random forests. Thus, the statistical power of the random-forests-based test could potentially be further enhanced by conducting proper hyperparameter tuning.

## Conclusion

As the simulation study showed, both proposed bagging-based testing procedures control the type I error, making them valid statistical testing procedures. Moreover, the bagging-based procedures induce a high statistical power for detecting GxE interactions compared to established GxE interaction tests. The novel random-forests-based test was the best GxE interaction testing method among all evaluated tests in many scenarios. In the real data application, the random-forests-based test detected a statistically significant GxE interaction regarding rheumatoid arthritis using $\textrm{NO}_2$ exposure as the environmental variable.

## Supplementary Information

Supplementary Information.

## Data Availability

The simulated data sets analyzed in this article are available from the corresponding author on reasonable request.

## Code availability

The proposed methods are implemented and publicly available in the R^31^ package GRSxE on CRAN.
