# Supplementary material for: Efficient gene–environment interaction testing through bootstrap aggregating
Source: Sci Rep. 2023 Jan 17;13:937. doi: 10.1038/s41598-023-28172-4 (PMC9845231; doi:10.1038/s41598-023-28172-4)
Supplement: Supplementary file 1 — Supplementary Information. [file 41598_2023_28172_MOESM1_ESM.pdf]

Additional file 1

Efficient gene-environment interaction testing through bootstrap aggregating

Michael Lau[1,2,*], Sara Kress[2], Tamara Schikowski[2] and Holger Schwender[1]

[1] *Mathematical Institute, Heinrich Heine University, Düsseldorf, Germany*
[2] *IUF – Leibniz Research Institute for Environmental Medicine, Düsseldorf, Germany*
[*] *Correspondence: michael.lau@hhu.de*

rsID	CHROM	POS	REF	ALT	MAF	TYPE	R2	ER2
rs9267989	6	32219320	G	T	0.178	IMPUTED	0.999	
rs9268145	6	32257284	T	G	0.194	GENOTYPED	1.000	0.995
rs522254	6	32273060	A	G	0.194	IMPUTED	0.994	
rs6910071	6	32282854	A	G	0.194	GENOTYPED	0.999	0.982
rs28361060	6	32303848	G	A	0.193	GENOTYPED	0.999	0.964
rs9268362	6	32333341	A	G	0.193	GENOTYPED	0.998	0.965
rs2073044	6	32338986	C	T	0.253	GENOTYPED	0.993	0.944
rs9268433	6	32345891	T	G	0.208	IMPUTED	0.983	
rs9268451	6	32349086	T	C	0.208	IMPUTED	0.983	
rs9268455	6	32349772	C	T	0.208	IMPUTED	0.983	
rs3793127	6	32371915	C	T	0.209	GENOTYPED	1.000	1.000
rs3763309	6	32375973	C	A	0.209	GENOTYPED	1.000	1.000
rs3763312	6	32376348	G	A	0.209	IMPUTED	0.999	
rs9268515	6	32379295	G	C	0.169	GENOTYPED	1.000	0.985
rs9268521	6	32381374	G	C	0.220	IMPUTED	0.996	
rs9268522	6	32381443	A	T	0.221	IMPUTED	0.995	
rs9268543	6	32384801	A	T	0.154	IMPUTED	0.997	
rs2395163	6	32387809	T	C	0.207	GENOTYPED	1.000	0.994
rs9268581	6	32396930	G	A	0.206	IMPUTED	0.996	
rs9268614	6	32402778	T	G	0.206	GENOTYPED	1.000	1.000
rs2395175	6	32405026	G	A	0.153	GENOTYPED	1.000	0.993
rs9268627	6	32405821	T	C	0.206	IMPUTED	0.999	
rs9268926	6	32433067	A	G	0.185	IMPUTED	0.985	
rs369515426	6	32542282	T	G	0.182	IMPUTED	0.683	
rs113322920	6	32553849	T	C	0.152	IMPUTED	0.794	
rs34855541	6	32559825	A	G	0.142	GENOTYPED	1.000	0.995
rs36096565	6	32560025	A	G	0.159	IMPUTED	0.850	
rs35395738	6	32560209	T	C	0.144	IMPUTED	0.900	
rs34415150	6	32560477	A	G	0.137	IMPUTED	0.849	
rs35118762	6	32560631	C	T	0.142	IMPUTED	0.986	
rs34928543	6	32560695	G	C	0.142	IMPUTED	0.987	
rs35265698	6	32561334	C	G	0.142	IMPUTED	0.984	
rs34350244	6	32561465	C	T	0.142	IMPUTED	0.944	
rs35294087	6	32561466	A	G	0.142	IMPUTED	0.977	
rs34553045	6	32561565	T	C	0.142	IMPUTED	0.941	
rs35371668	6	32561638	C	T	0.139	IMPUTED	0.889	
rs34647096	6	32561681	G	A	0.142	IMPUTED	0.977	
rs188575117	6	32561935	A	C	0.142	IMPUTED	0.923	
rs2760985	6	32566398	G	A	0.142	IMPUTED	0.978	
rs687308	6	32567256	C	T	0.142	IMPUTED	0.980	
rs35117964	6	32568146	A	G	0.151	IMPUTED	0.924	
rs34039593	6	32570311	T	G	0.142	GENOTYPED	1.000	1.000
rs2647066	6	32571122	C	T	0.142	IMPUTED	0.973	

rs17425622	6	32571961	T	C	0.142	IMPUTED	0.950	
rs601945	6	32573415	A	G	0.142	IMPUTED	0.967	
rs602457	6	32573562	T	C	0.142	IMPUTED	0.939	
rs7760841	6	32574868	C	T	0.138	IMPUTED	0.944	
rs560530	6	32577222	G	A	0.183	IMPUTED	0.984	
rs660895	6	32577380	A	G	0.183	GENOTYPED	1.000	0.999
rs532965	6	32577973	T	G	0.142	IMPUTED	0.997	
rs3997868	6	32578590	A	G	0.183	IMPUTED	0.992	
rs3997872	6	32580617	T	A	0.142	IMPUTED	0.996	
rs521539	6	32581973	G	A	0.183	GENOTYPED	1.000	1.000
rs3129751	6	32582189	A	C	0.142	IMPUTED	0.999	
rs3104415	6	32582577	A	C	0.324	IMPUTED	0.998	
rs34656207	6	32582601	C	T	0.346	IMPUTED	0.826	
rs3104413	6	32582650	C	G	0.142	GENOTYPED	0.999	0.972
rs3129754	6	32583046	A	G	0.408	IMPUTED	0.948	
rs3129756	6	32583063	A	G	0.376	IMPUTED	0.801	
rs6605556	6	32583099	A	G	0.142	IMPUTED	0.939	
rs4959106	6	32583159	T	C	0.430	GENOTYPED	1.000	0.996
rs6931044	6	32583194	G	T	0.408	IMPUTED	0.997	
rs34850435	6	32583299	C	T	0.431	IMPUTED	0.991	
rs6931277	6	32583357	A	T	0.142	GENOTYPED	1.000	0.993
rs6941972	6	32583529	G	A	0.032	IMPUTED	0.455	
rs36124427	6	32583677	T	C	0.430	IMPUTED	0.997	
rs1281935	6	32583820	G	T	0.050	IMPUTED	0.974	
rs34028938	6	32584346	C	A	0.430	IMPUTED	0.990	
rs510205	6	32584693	C	G	0.183	IMPUTED	0.985	
rs1281931	6	32587966	T	C	0.051	GENOTYPED	0.996	0.851
rs9271608	6	32591588	A	G	0.142	IMPUTED	0.989	
rs3104375	6	32600101	G	C	0.142	IMPUTED	0.953	
rs1391371	6	32603798	A	T	0.155	IMPUTED	0.917	
rs9272417	6	32605078	A	G	0.143	IMPUTED	0.934	
rs9272461	6	32605609	G	A	0.149	IMPUTED	0.951	
rs41269945	6	32607853	A	T	0.140	IMPUTED	0.873	
rs17426593	6	32608077	T	C	0.142	IMPUTED	0.873	
rs41269955	6	32608269	G	A	0.137	IMPUTED	0.854	
rs34141382	6	32608478	T	C	0.130	IMPUTED	0.848	
rs34763586	6	32608998	T	C	0.140	IMPUTED	0.862	
rs34965214	6	32609545	C	T	0.142	IMPUTED	0.834	
rs9272785	6	32610401	G	A	0.142	IMPUTED	0.844	
rs28724243	6	32629347	T	C	0.305	IMPUTED	0.850	
rs9275222	6	32659516	A	T	0.461	IMPUTED	0.988	
rs4713582	6	32660051	T	C	0.496	GENOTYPED	1.000	1.000
rs9275511	6	32674329	G	A	0.456	IMPUTED	0.935	
rs7764856	6	32680640	T	A	0.326	GENOTYPED	0.999	0.984

Table S1: Information on the 87 SNPs analyzed in the real data application using the SALIA data set. rsID: Reference SNP cluster ID; CHROM: Chromosome; POS: Reference position; REF: Reference allele; ALT: Alternative non-reference allele; MAF: Minor allele frequency; TYPE: Variant genotyped or imputed; R2: Imputation quality (estimate of the squared correlation between imputed genotypes and true/unobserved genotypes); ER2: Empirical R^2 for genotyped variants (not calculated for imputed variants).

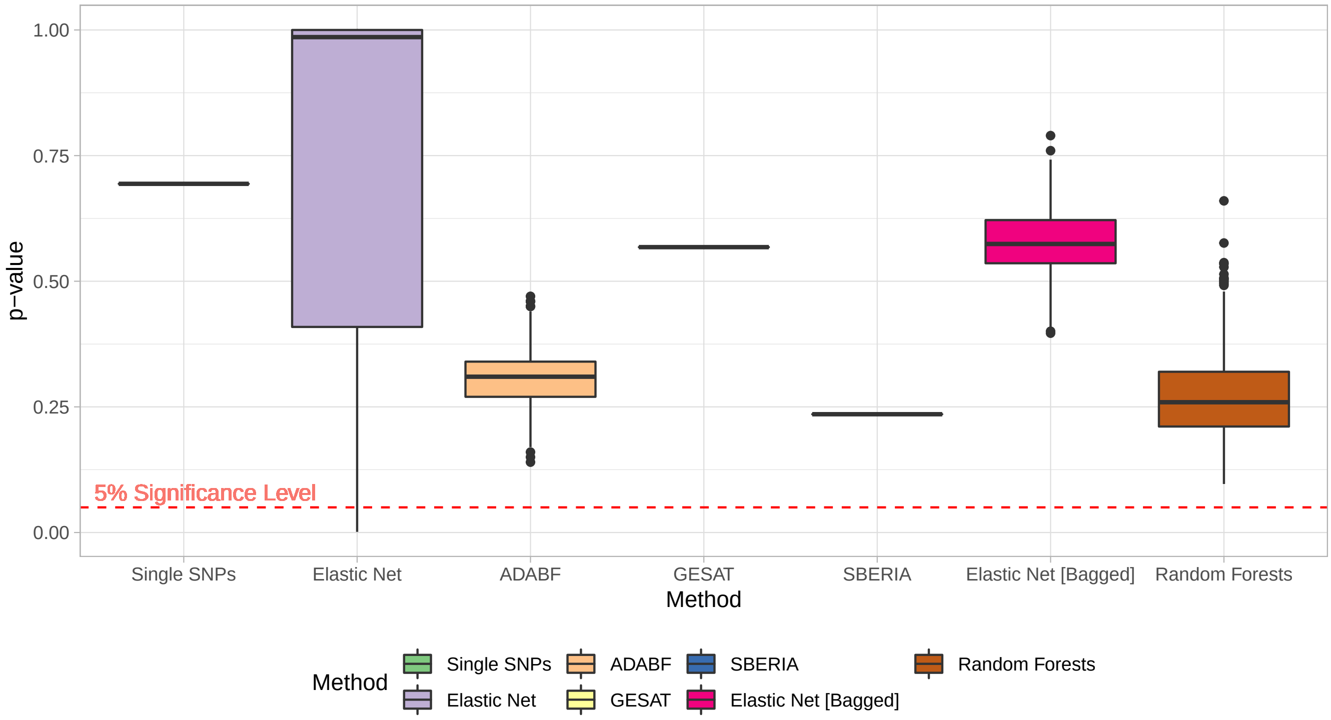

Figure S1: p-values of 1000 independent applications of the GxE interaction testing procedures to the considered real data set from the SALIA cohort study analyzing the gene-based SNP selection containing 72 SNPs. For elastic net, the train/test data splits changed. For the two bagging-based tests, the bootstrap samples changed. For ADABF, the random sampling from the null distribution of GxE interaction coefficients changed. The single-SNP-based test, GESAT, and SBERIA were applied only once, since there is no randomness involved in the application of these tests.

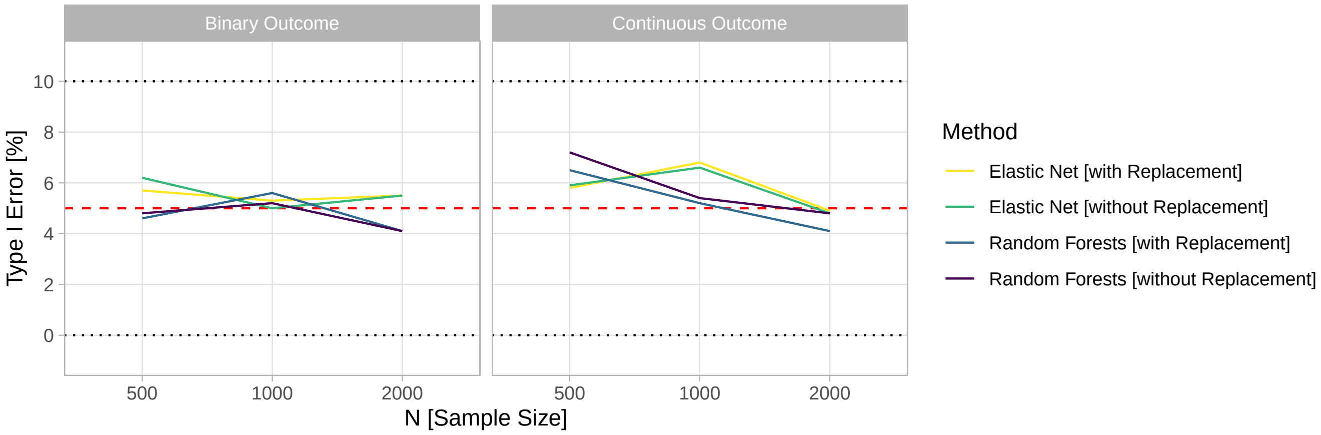

Figure S2: Type I error rates of the bagging-based GxE interaction tests using sampling with or without replacement in the simulation study

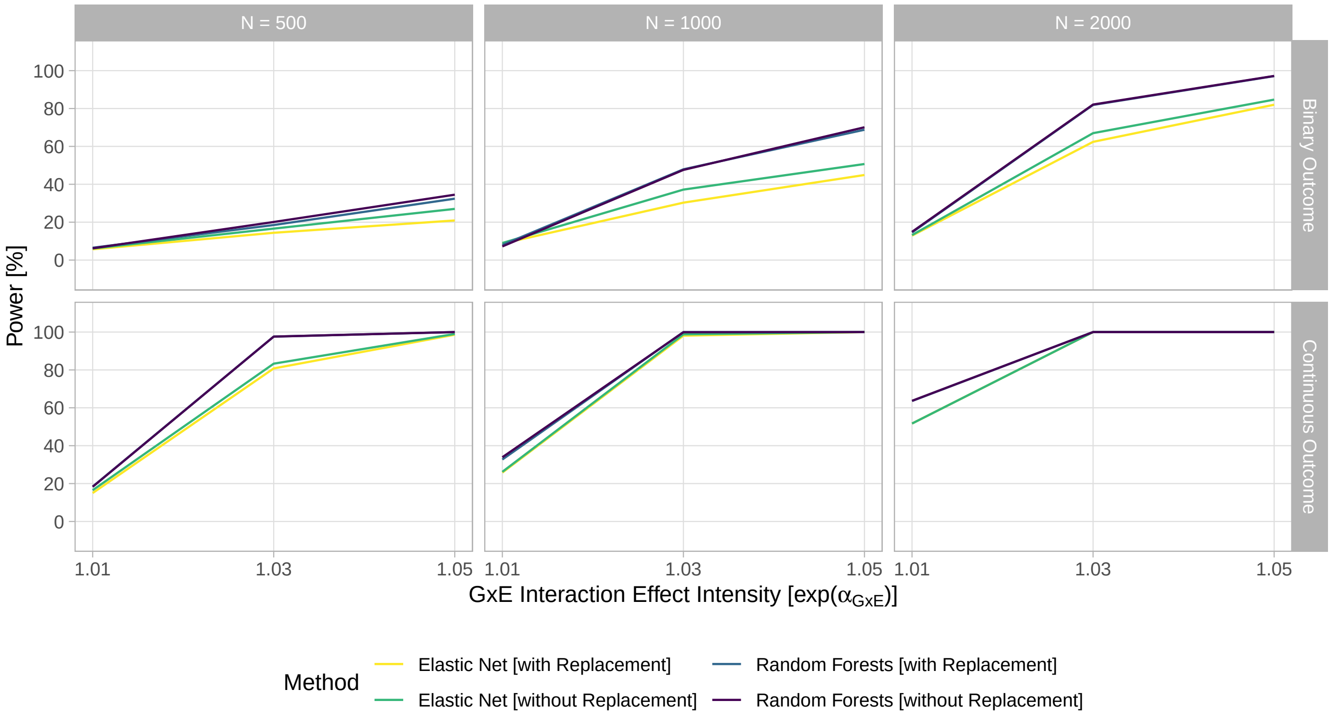

Figure S3: Power of the bagging-based GxE interaction tests using sampling with or without replacement in the simulation study

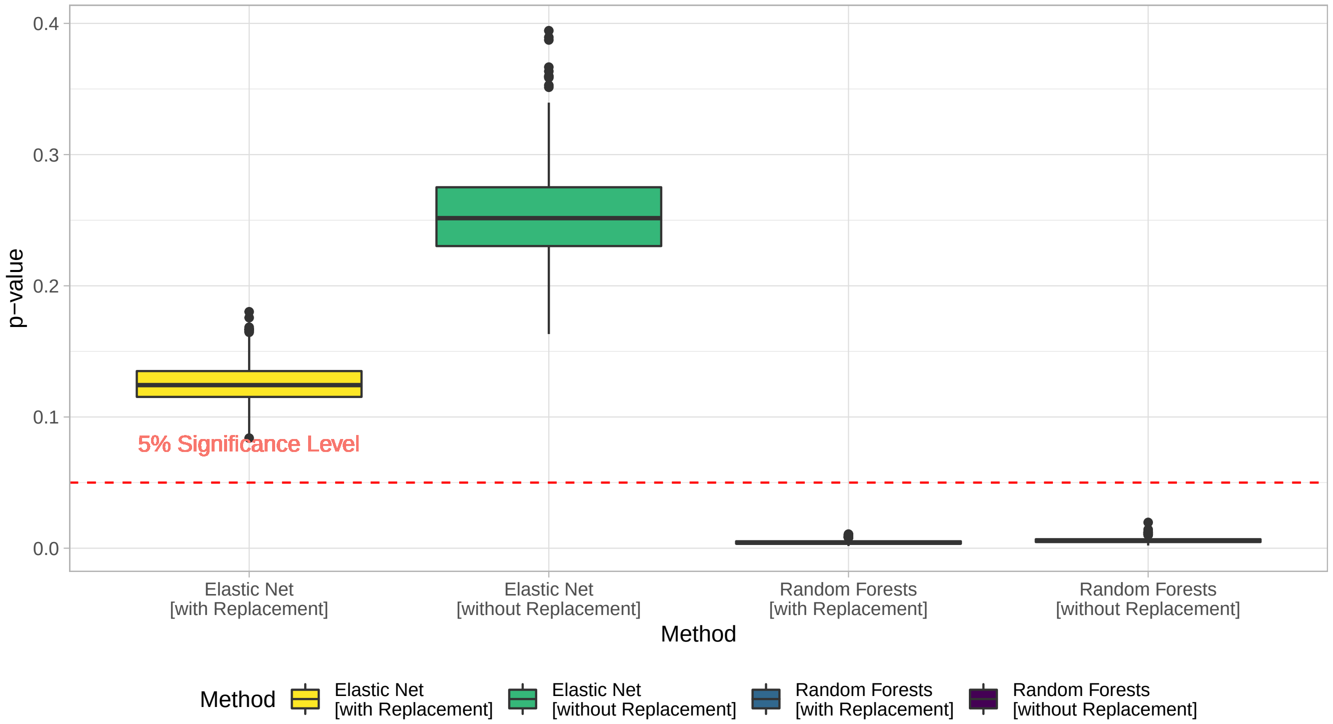

Figure S4: p-values of the bagging-based GxE interaction tests using sampling with or without replacement in the real data application
